# The Polymorphisms in *LNK* Gene Correlated to the Clinical Type of Myeloproliferative Neoplasms

**DOI:** 10.1371/journal.pone.0154183

**Published:** 2016-04-25

**Authors:** Yan Chen, Fang Fang, Yang Hu, Qian Liu, Dingfang Bu, Mei Tan, Liusong Wu, Ping Zhu

**Affiliations:** 1 Department of Hematology, Peking University First Hospital, Beijing, China; 2 Zunyi Medical College Affiliated Hospital, Zunyi, Guizhou, China; B.C. Cancer Agency, CANADA

## Abstract

**Objective:**

LNK is an adapter protein negatively regulating the JAK/STAT cell signaling pathway. In this study, we observed the correlation between variation in *LNK* gene and the clinical type of myeloproliferative neoplasms (MPN).

**Methods:**

A total of 285 MPN cases were recruited, including essential thrombocythemia (ET) 154 cases, polycythemia vera (PV) 76 cases, primary myelofibrosis (PMF) 19 cases, and chronic myeloid leukemia (CML) 36 cases. Ninety-three healthy individuals were used as normal controls. V617F mutation in JAK2 was identified by allele-specific PCR method, RT-PCR was used for the detection of *BCR/ABL1* fusion gene, and mutations and variations in coding exons and their flanking sequences of *LNK* gene were examined by PCR-sequencing.

**Results:**

Missense mutations of A300V, V402M, and R415H in LNK were found in 8 patients including ET (4 cases, all combined with *JAK2*-V617F mutation), PV (2 cases, one combined with *JAK2*-V617F mutation), PMF (one case, combined with *JAK2*-V617F mutation) and CML (one case, combined with *BCR/ABL1* fusion gene). The genotype and allele frequencies of the three SNPs (rs3184504, rs111340708 and rs78894077) in *LNK* were significantly different between MPN patients and controls. For rs3184504 (T/C, in exon2), the T allele (p.262W) and TT genotype were frequently seen in ET, PV and PMF (P<0.01), and C allele (p.262R) and CC genotype were frequently seen in CML (P<0.01). For rs78894077 (T/C, in exon1), the T allele (p.242S) was frequently found in ET (P<0.05). For rs111340708 (TGGGGx5/TGGGGx4, in intron 5), the TGGGG x4 allele was infrequently found in ET, PMF and CML(P<0.01).

**Conclusion:**

Mutations in *LNK* could be found in some of MPN patients in the presence or absence of *JAK2*-V617F mutation. Several polymorphisms in *LNK* gene may affect the clinical type or the genetic predisposition of MPN.

## Introduction

Myeloproliferative neoplasms (MPN) area group of diseases characterized by unrestricted proliferation of hematopoietic stem cells or progenitor cells, which usually include polycythemia vera (PV), essential thrombocythemia (ET), primary myelofibrosis (PMF) and chronic myeloid leukemia (CML). They have the tendency to progress to acute leukemia with a worse prognosis. In 2005, several groups identified V617F mutation in *JAK2* gene (*JAK2*-V617F) in patients with PV, ET and PMF[1–4]. More than 95% PV patients are *JAK2*-V617Fpositive, while approximately 50–60% ET and PMF patients carry the mutation[5, 6]. In addition, mutations in exon 12 of *JAK2* are identified in PV patients with or without*JAK2*-V617F mutation[7, 8], and W515L/K mutations in *MPL* gene are detected in ET and PMF patients with or without *JAK2*-V617F mutation[9, 10]. 95% CML patients have the Ph chromosome, which contains a *BCR/ABL1* translocated gene encoding a BCR/ABL1 fusion protein. This fusion protein has a sustained tyrosine kinase activity that phosphorylates the tyrosine residues in its molecule and in the downstream substrates, through which JAK/STAT signaling is abnormally activated resulting in the inhibition of apoptosis, transformation of cells and proliferation of malignant clones. The medicine Imatinib targets the fusion protein, and is currently the most important therapy for CML patients with *BCR/ABL1* fusion gene. The presence of *JAK2*-V617F mutation in PV, ET and PMF and *BCR/ABL1* fusion gene in CML have become the important indicators for clinical diagnosis of these diseases. However, the molecular bases in MPN patients without *JAK2*-V617F mutation and *BCR/ABL1* fusion gene remain unknown.

We recently studied the clonal evolution of an ET patient without *JAK2*-V617F mutation using the single-cell exome sequencing technique, and found that several other regulatory factor genes such as *SESN2* and *NTRK1* may be involved in the neoplasm progression[11]. Several evidences have indicated that mutations in other molecules in the signaling pathways relating to cellular development especially in JAK2/STAT signaling pathways are also involved in the pathogenesis of MPN. LNK (SH2B3) is an adapter protein negatively regulating cytokine signaling through interaction with MPL and JAK2 to inhibit the downstream molecule STAT5 [12–14]. LNK gene and the SNP rs3184504 in LNK are closely related to immunological responses, diabetes, and LDL-cholesterol level [15]. LNK has extensive roles in myeloid development and hematopoiesis. For example, *LNK*-/- mice are very sensitive to cytokine stimulation, and display leukocytosis, thrombocythemia and splenomegaly [15, 16], similar to the phenotype of MPN[12, 17]. Megakaryocytic hyperplasia is also observed in spleen and bone marrow in *LNK* -/- mice [12, 17]. LNK regulates the renewal of hematopoietic stem cells, and controls their proliferation [18–20]. LNK binds to phosphorylated JAK2 upon thrombopoietin stimulation with higher affinity, forming a negative feedback to prevent JAK2/STAT signaling from being hyperactivated. *LNK* mutations may represent the early genetic events in pathogenesis of MPN, similar to *JAK2*-V617F in PV [19, 20]. In a screening of 33 *JAK2*-V617F negative patients with ET or PMF, Oh et al. identified two individuals with mutations in exon2 of *LNK*[19]. One PMF patient exhibited a five base-pair deletion and missense mutation leading to loss of the pleckstrin homology (PH) and SH2 domains, and the second ET patient had a missense mutation (E208Q) in PH domain. Some MPN patients specifically acquire *LNK* mutations after chronic blast phase transformation [21], suggesting that the *LNK* mutations may play roles in the pleiotropic transformation of MPN. The changes of LNK function due to mutations or single nucleotide polymorphisms (SNP) may affect the JAK2/STAT signaling pathways, leading to the pathogenesis or changing the phenotype of MPN. Here we recruited 285 MPN patients including ET, PV, PMF and CML to search for mutations in *LNK* and to examine the genotypes of several SNPs in *LNK*, and analyzed the relationship between the genotypes of several SNPs and the phenotype of MPN in these patients.

## Patients and Methods

### Patients

We recruited 285 patients with MPN (males 133 cases and females 152 cases) including CML 36 cases, PV 76 cases, ET154 cases, and PMF 19 cases with an average age of 53.2 years old (8–73 years old) from Hematology Department of Peking University First Hospital and Beijing Dopei Hospital. The diagnosis of MPN was based on the criteria from WHO Classification of Tumors of Hematopoietic and Lymphoid Tissues, 2008. Ninety-three healthy people were also included as normal controls (males 53 cases and females 40 cases, with an age range of 20–53 years and an average age of 38.8 years). Bone marrow or peripheral blood was used to extract genomic DNA by a genomic DNA extraction kit (Tian Yi, Beijing, China). Written informed consent was obtained from the patients and healthy controls. If the participant is under 18, written informed consent was obtained from the parents or legal guardians. The investigation protocol was approved by the Medical Ethics Committee of Peking University First Hospital.

### Detection of JAK2-V617F mutation in JAK2 and BCR/ABL1 fusion gene

*JAK2*-V617F mutation in genomic DNA samples was detected by allele-specific PCR method and confirmed by PCR-sequencing[22]. We used reverse transcription and multiplex PCR to identify *BCR/ABL1* fusion gene in total blood RNA samples [23].

### Detection of mutations and genotyping of 3 SNPs in LNK gene

Genomic DNA samples were used to amplify a part of the coding exon 1 and coding exons 2-6of *LNK* using 3 pairs of primers: 1-F: 5’-CGGAGAGGCTGCTGAGAC and 1-R: 5’-TTGCACTCGGCCTAAAAGTT; 2-4-F:5’-AACTCAGGCCTGGCTGG and 2-4-R: 5’-GGGCTACCTTATGTCCTGGG; 5-6-F: 5’-GTACGCTGGAACCCAGACTC and 5-6-R:5’-GTCTGCAGCAAGCCTCTACC. PCR products were purified and sequenced on an ABI PRISM377 sequencer. Three important SNPs including rs78894077 (in exon 1), rs3184504 (in exon 2), and rs11340708 (in intron 5) were encompassed in the PCR products, and their genotypes can be identified by sequencing.

### Statistical analysis

The genotype frequency of the SNPs was test for Hardy-Weinberg equilibrium using the HWE software. Chi-square test or Fisher’s exact test were used for the comparison of numerical data among groups.

## Results

### 1. BCR/ABL1 fusion gene and JAK2-V617F mutation in the 285 MPN patients

The 36 CML cases were positive for *BCR/ABL1* fusion gene. For *JAK2*-V617F mutation, 69 of 76 PV cases, 70 of 154 ET cases, and 8 of 19 PMF cases were positive. All control samples were negative for *BCR/ABL1* fusion gene and *JAK2*-V617F mutation.

### 2. The allele frequencies of the SNPs rs78894077, rs3184504 and rs11340708 in normal Chinese controls of Han nationality

In the PCR products amplified from exons 1–6 of *LNK* gene, the sites of the 3 SNPs (rs78894077 in exon 1, rs3184504 in exon 2, and rs11340708 in intron 5) were included and the genotypes of the 3 SNPs could be identified as well. The allele frequencies of rs78894077, rs3184504, and rs11340708 in normal controls were C 0.097/T 0.903, C 0.5/T 0.5, and TGGGG x5 0.462/ TGGGG x4 0.538, respectively.

### 3. Missense mutations were detected in 8 of the 285 MPN patients, including homozygous A300V mutation found in 3 ET cases with JAK2-V617F mutation

**Fig 1:**
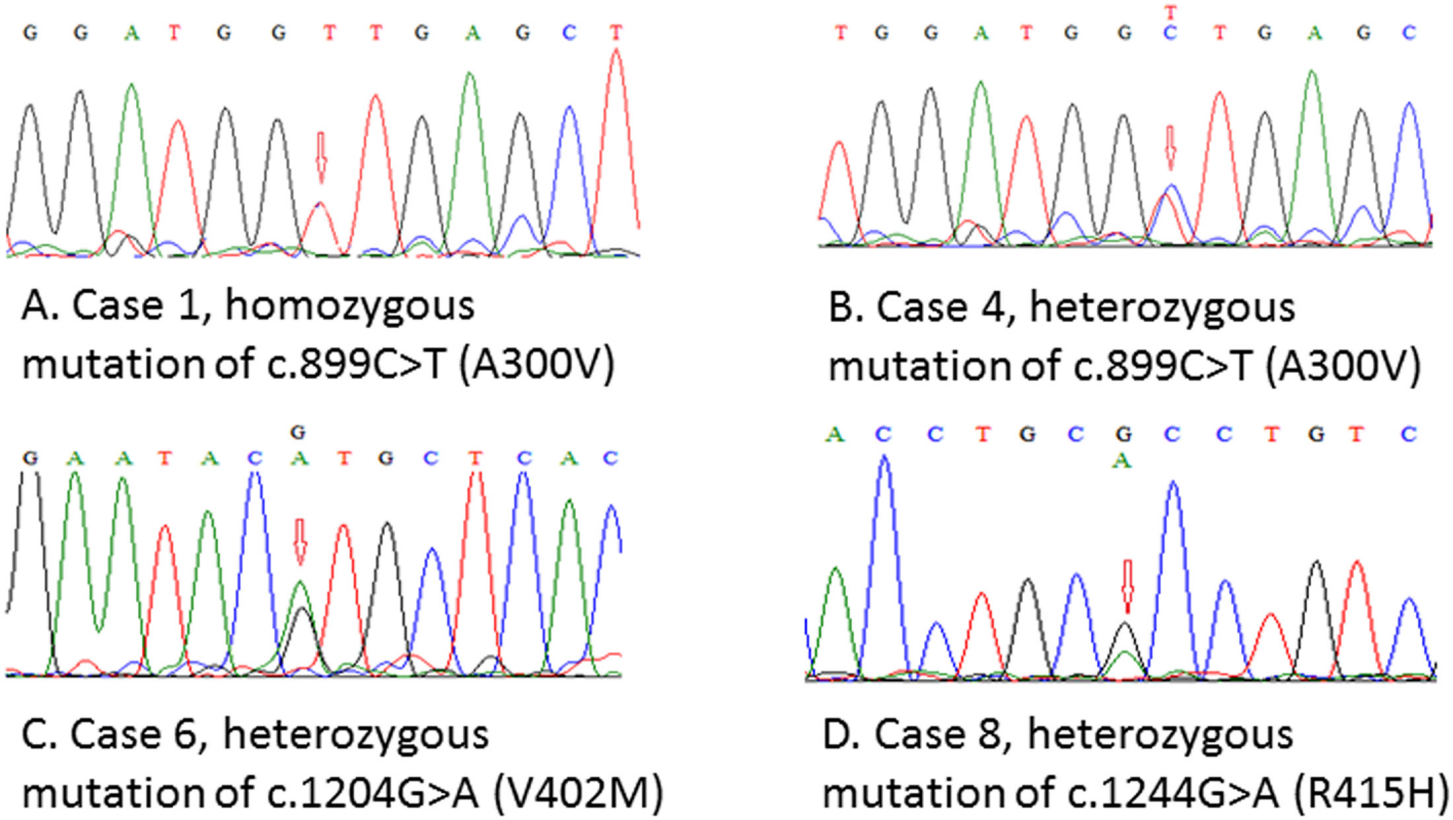
Sequencing results showing the mutations found in MPN cases. A. Case1, homozygous mutation of c.899C>T (A300V). B. Case4, heterozygous mutation of c.899 C>T (A300V). C. Case6, heterozygous mutation of c.1204 G>A (V402M). D. Case8, heterozygous mutation of c. 1244 G>A (R415H).

The 8 patients and their mutations in *LNK* are shown in Table 1 and Fig 1. Six of them also carried *JAK2*-V617F mutation. The functional effects of the 3 amino acid substitutions were predicted by PolyPhen-2 (http://genetics.bwh.harvard.edu/pph2/). All belong to “possible damage” changes, and the 3 amino acid residues in wild-type proteins are highly conservative in organisms. We also searched for SNP database (http://www.ncbi.nlm.nih.gov/snp/).c.1204G>A (V402M) is recorded as rs755796482 with the MAF of A = 0.000, and this amino acid substitution has been reported in Ph-like acute lymphoblastic leukemia [24]; c.899C>T (A300V) and c.1244G>A (R415H) are not found in the SNP database.

**Table 1:** Mutations in *LNK* gene found in 8 of the 285 MPN cases.

Case no.	Sex/age (year)	Disease	*JAK2*-V617F mutation	*LNK*mutation	Heterozygous/homozygous mutation	LNK domain involved
1	F/72	ET	+	c.899C>T (A300V)	Homo.	PH
2	F/51	ET	+	c.899C>T (A300V)	Homo.	PH
3	M/57	ET	+	c.899C>T (A300V)	Homo.	PH
4	M/58	PV	-	c.899C>T (A300V)	Hetero.	PH
5	M/38	CML	-,(*BCR/ABL1*+)	c.899C>T (A300V)	Hetero.	PH
6	F/68	ET	+	c.1204G>A (V402M)	Hetero.	SH2
7	F/46	PMF	+	c.1204G>A (V402M)	Hetero.	SH2
8	M/48	PV	+	c.1244G>A (R415H)	Hetero.	SH2

### 4. Polymorphism of SNP rs3184504 changes a codon, c.784T (p.262W) was frequently found in ET, PV and PMF, and c.784C (p.262R) was frequently seen in CML

**Fig 2:**
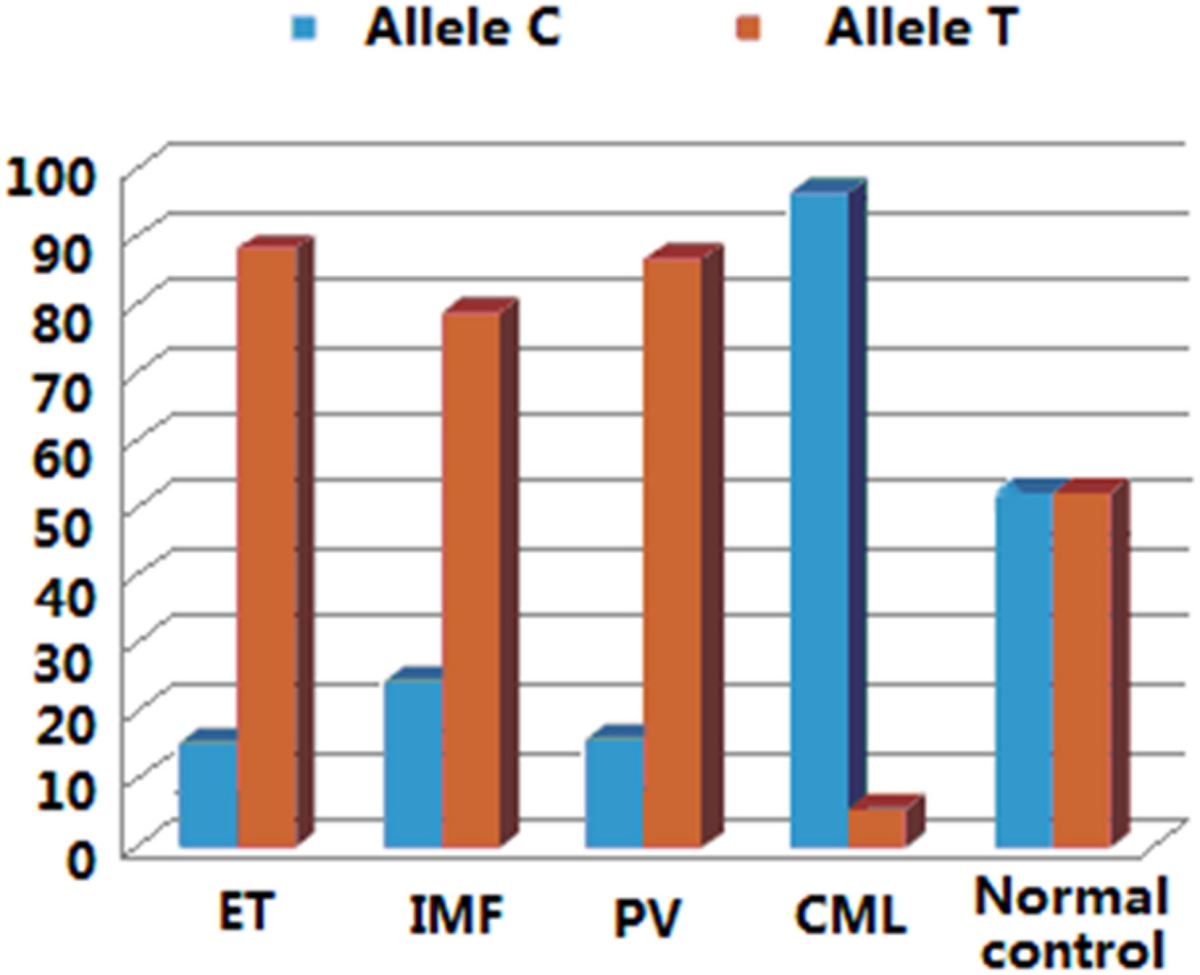
Allele frequencies of rs3184504 in MPN patients. The T allele frequency was 88.3%, 86.8% and 78.9% in ET, PV and PMF, respectively, significantly higher than that in normal controls (50.0%; P<0.01). The C allele frequency was 94.4% in CML, higher than that in normal controls (50.0%, P<0.01).

SNP rs3184504 is the polymorphism of T/C at c.784 in coding exon 2 of *LNK* gene. The C allele makes the codon CGG (p.262R) and the T allele constitutes the codon TGG (p.262W). The genotype frequencies CC, CT and TT of this SNP were significantly different between normal controls and the MPN patients of ET, PV, PMF and CML. The T allele frequency was 88.3%, 86.8% and 78.9% in ET, PV and PMF, respectively, significantly higher than that in normal controls (50.0%; *P*<0.01). When the MPN patients have *JAK2*-V617F or other mutations, p.262W in LNK due to T allele of rs3184504 may facilitate the development of MPN to the phenotype of ET, PV or PMF. In contrast, the C allele (p.262R) frequency was 94.4% in CML, much higher than that in normal controls (50.0%, *P*<0.01). Compared to the patients with p.262W (T allele of rs3184504) in LNK, those with p.262R(C allele of rs3184504) are at a higher risk to develop CML (*P*<0.01; OR = 17, 95%CI = 5.957–48.510). Therefore, the allele type of rs3184504 may influence the pathogenesis of MPN (Fig 2 and Table 2).

**Table 2:** Genotype frequency and allele frequency of rs3184504 in MPN patients.

Group	Number	Genotype frequency Number (%)				Allele frequency Number(%)		
		CC	CT	TT	P value*	C	T	P value*
ET	154	18(11.7)	0(0.0)	136(88.3)	<0.001	36(11.7)	272(88.3)	<0.001
PV	76	10(13.2)	0(0.0)	66(86.8)	<0.001	20(13.2)	132(86.8)	<0.001
PMF	19	4(21.1)	0(0.0)	15(78.9)	<0.001	8(21.1)	30(78.9)	0.001
CML	36	34(94.4)	0(0.0)	2(5.6)	<0.001	68(94.4)	4(5.6)	<0.001
Normal	93	38(40.9)	17(18.3)	38(40.9)		93(50.0)	93(50.0)	

*: Fisher exact test, compared to normal group.

### 5. Polymorphism of SNP rs78894077 changes a codon, c.724C (p.242P) was frequently seen in CML, while c.724T (p.242S) was frequently found in ET

**Fig 3:**
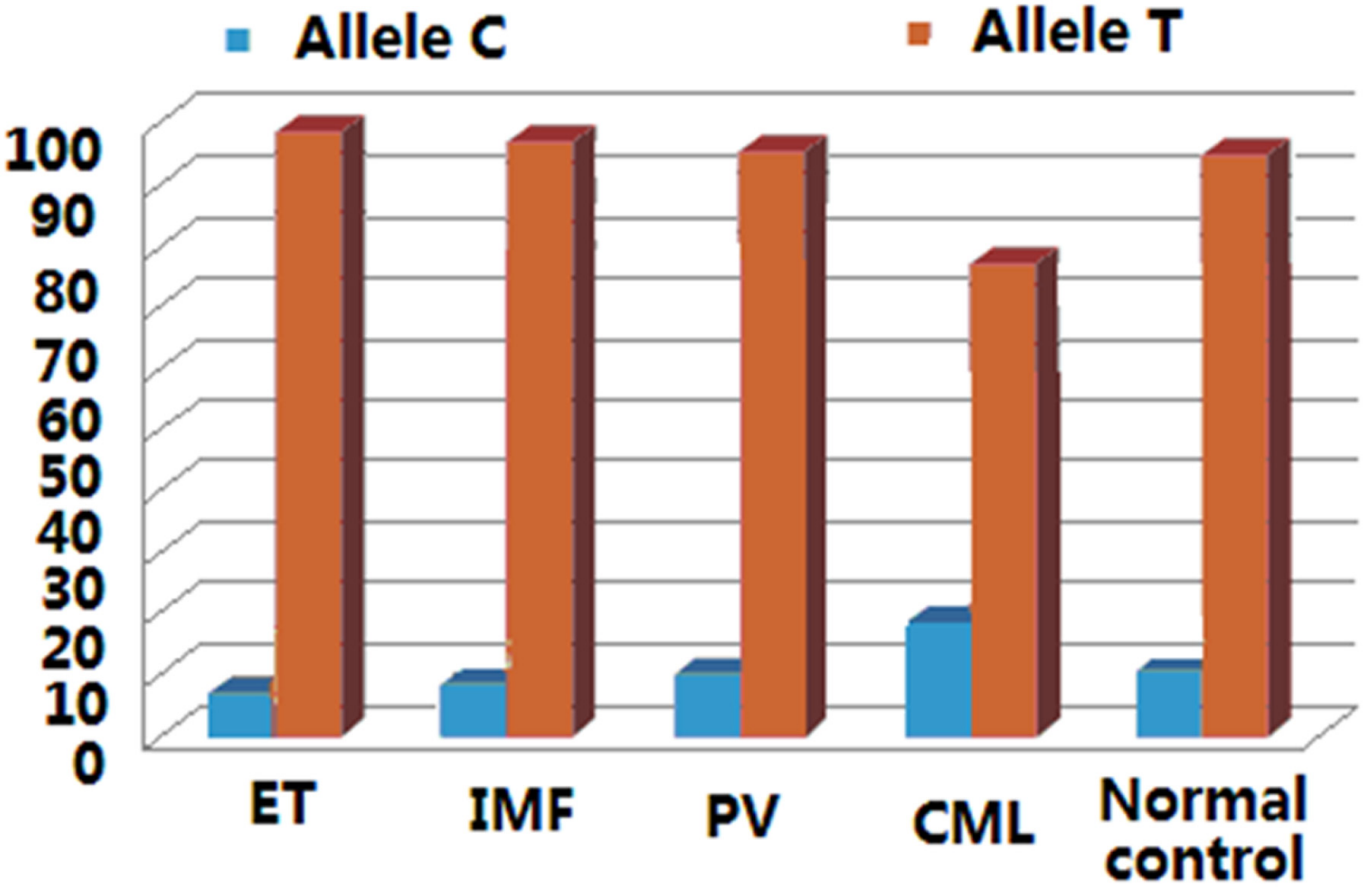
Allele frequencies of rs78894077 in MPN patients. The T allele frequency was 95.5%, 91.4%, 94.7% and 90.3% in ET, PV, PMF and normal controls respectively. The T allele frequency (p.242S) is relatively higher in ET than in normal controls (P = 0.025).

SNP rs78894077 locates in exon 1 and near the 3’ end of exon 1. c.724C makes the codon CCA (p.242P), and c.724T makes the codon TCA (p.242S). The genotype frequency is different between normal controls and CML with relatively more CC and less TC genotypes in CML (*P*<0.001). The T allele frequency is relatively higher in ET than in normal controls (*P* = 0.025), suggesting that p.242S is frequently seen in MPN patients with the phenotype of ET (Fig 3 and Table 3).

**Table 3:** Genotype frequency and allele frequency of rs78894077 in MPN patients.

Group	Number	Genotype frequency Number (%)				Allele frequency Number (%)		
		TT	TC	CC	P value*	T	C	P value*
ET	154	140(90.9)	14(9.1)	0(0.0)	0.069	294(95.5)	14(4.5)	0.025
PV	76	63(82.9)	13(17.1)	0(00.0)	0.662	139(91.4)	13(8.6)	0.722
PMF	19	17(89.5)	2(10.5)	0(0.0)	0.685	36(94.7)	2(5.3)	0.385
CML	36	30(83.3)	0(0.0)	6(16.7)	<0.001	60(83.3)	12(16.7)	0.116
Normal	93	76(81.7)	16(17.2)	1(1.1)		168(90.3)	18(9.7)	

*: Fisher exact test, compared to normal group.

### 6. SNP rs111340708 is a polymorphism of repeated sequence in intron 5, and the allele frequency of TGGGG x4 was infrequently found in ET, PMF and CML

**Fig 4:**
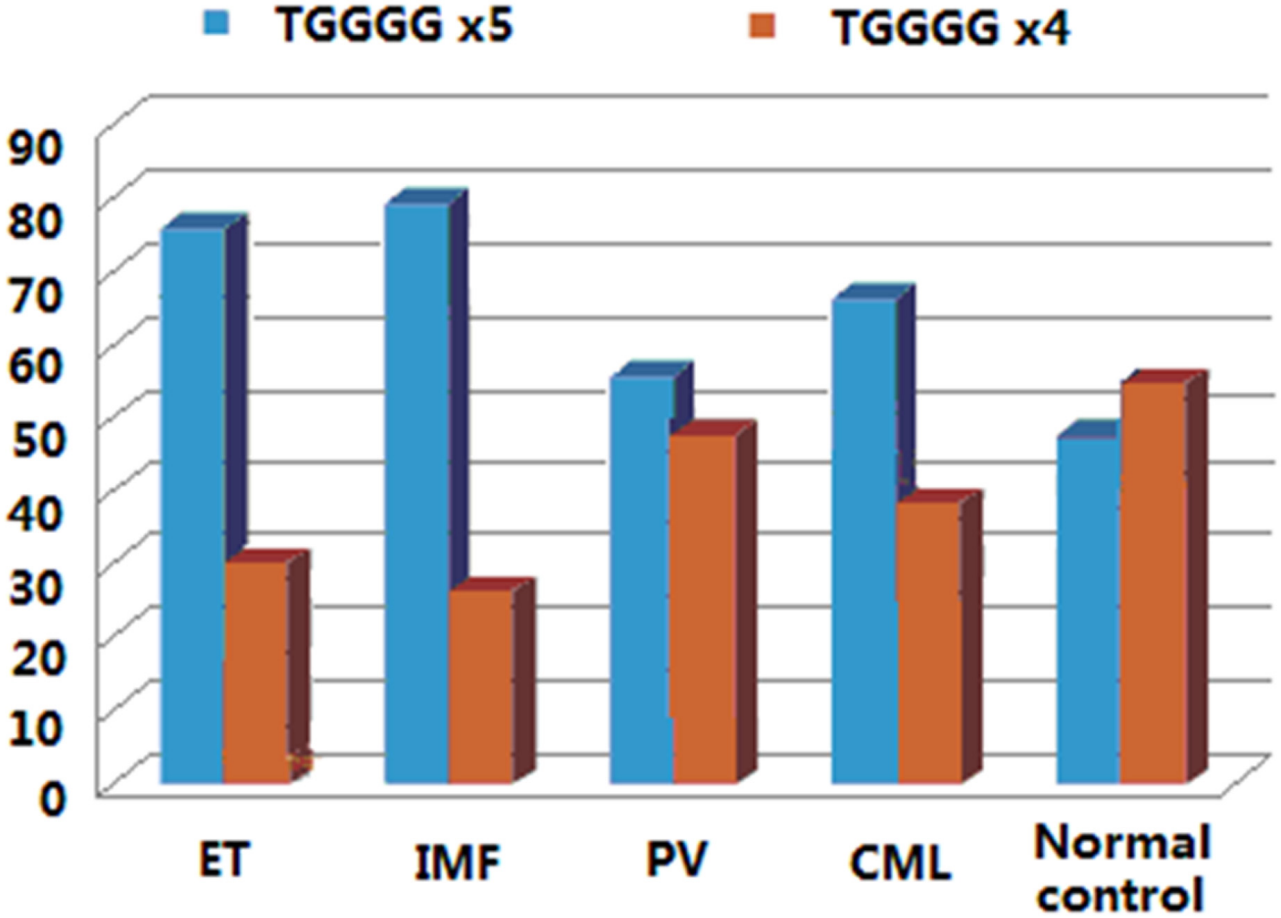
Allele frequencies of rs111340708 in MPN patients. The allele frequency of TGGGG x4 was 28.1%, 21.1%, and 36.1% in ET, PMF and CML patients respectively, which were lower than those in normal controls (53.8%, P<0.01).

SNP rs111340708 is a polymorphism of the repeated sequence of TGGGG (TGGGG x5/TGGGG x4), which locates in intron 5 near the donor site of intron 5 (c.1236+4~28). The genotype frequency was different between normal controls and MPN patients of ET, PV, PMF and CML, with less genotype of TGGGG x4/TGGGG x4 in these patients (*P*< 0.01). The allele frequency of TGGGG x4 was also lower in ET, PMF and CML patients than in normal controls (*P*<0.01; Fig 4 and Table 4).

**Table 4:** Genotype frequency and allele frequency of rs111340708 in MPN patients.

Group	Number	Genotype frequency Number (%)				Allele frequency Number (%)		
		TGGGG x5	TGGGG x5/x4	TGGGG x4	P value*	TGGGG x5	TGGGG x4	P value*
ET	154	100(64.7)	22(14.4)	32(20.9)	<0.001	222(72)	86(28)	<0.001
PV	76	28(36.8)	25(32.9)	23(30.3)	0.001	81(53.3)	71(46.7)	0.197
PMF	19	13(68.4)	4(21.1)	2(10.5)	0.009	30(78.9)	8(21.1)	<0.001
CML	36	18(50.0)	10(27.8)	8(22.2)	0.008	46(63.9)	26(36.1)	0.011
Normal	93	38(40.9)	10(10.7)	45(48.4)		86(46.2)	100(53.8)	

*: Fisher exact test, compared to normal group.

## Discussion

MPN include ET, PV, PMF and CML which origin from the uncontrolled proliferation of a cell lineage in bone marrow. Previous studies have demonstrated that mutations in *LNK* gene can lead to MPN of various clinical types. To investigate the relationship between variations in *LNK* gene and the clinical type of MPN, we examined mutations and genotypes of several SNPs in *LNK* in a cohort of MPN patients. We found that the genotype frequency and allele frequency of SNPs rs3184504 (C/T) was closely related to the clinical types of MPN. p.262W in *LNK* due to the T allele of rs3184504 was frequently found in MPN patients with the clinical type of ET, PV and PMF, while p.262R due to the C allele of rs3184504 was frequently seen in CML. The *LNK* variation that affects disease sensitivity has been mainly focused on rs3184504 in the literature. The genotype of rs3184504 has been reported to relate to platelet number [25], hemoglobin concentration and hematocrit[26]. Recently, studies have also found that the genotype of rs3184504 may be involved in many diseases including blood hypertension[27, 28], eosinophil number[29], myocardial infarction, type I diabetes[15], hyper-LDL cholesterolemia[16], and the high concentration of soluble ICAM-1 that can lead to subclinical atherosclerosis[30]. p.262W due to the T allele of rs3184504 was related to celiac disease[31]. However, genetic variations in *LNK* relating to the phenotype of MPN have not been reported to date. Our findings that p.262W frequently seen in ET, PV and PMF, and p.262R frequently found in CML (OR = 17) suggest the importance of p.262 variation in determining the clinical type of MPN. MPN patients with p.262W have the tendency to affect megakaryocyte, erythrocyte or fibroblast in hematopoietic tissue, while those with p.262R are likely to develop abnormalities in myeloid system (CML). In other words, Trp or Arg at p.262 in LNK may have influences on the malignant differentiation of hematopoietic cells in bone marrow.

The SNP rs78894077 locates in coding exon 1 and near the 3’ end of exon 1. This polymorphism changes codon 242 in *LNK*. MPN patients with T allele (p.242S) were frequently observed in ET patients, and p.242S may therefore exert a genetic predisposition for megakaryocyte proliferation and ET. The SNP rs111340708 is a splice region variant of repeated sequence in intron 5. The allele type of TGGGG x4 was relatively uncommon in ET, PMF and CML. Therefore, the genotype and allele frequencies of rs78894077 and rs111340708 may also have the effects on the malignant proliferation of hematopoietic cells in bone marrow.

We found 3 kinds of missense mutations (A300V, V402M and R415H) located in PH or SH2 domain in LNK in 8 MPN patients, of whom 6 had *JAK2*-V617F mutation as well. A previous study has reported the detection of 2 somatic mutations in *LNK* from 33 *JAK2*-V627F negative MPN samples [19]. In a study on mutations in *TET2*,*IDH*, *JAK2* and *MPL1* in 78 MPN cases no *LNK* mutation was detected, demonstrating that the coexistence of *LNK* mutations and other MPN-related gene mutations was rare[23]. In contrast, we found the coexistence of *JAK2*-V617 mutation and *LNK* mutation in 6 of the 8 MPN patients. We predict that the mutations we detected in the 8 patients (Table 1) are germline mutations. The genomic DNA samples we examined were from bone marrows or peripheral leukocytes that mixed with various cell subsets such as T lymphocytes, B lymphocytes, nucleated erythrocytes, and megakaryocytes. Therefore, homozygous mutations should indicate germline mutations (the 3 ET patients in Table 1). In the 5 patients carrying heterozygous mutations, sequencing traces at the heterozygous mutation sites show roughly equal height of the double peaks (Fig 1), suggesting the presence of germline mutations. Unfortunately, we could not get the DNA samples from non-hematological cells such as scratched buccal cells to confirm our prediction.PH domain and SH2 domain are critical for functioning of LNK, mutations in PH or SH2 domain result in a partial or a complete loss of inhibitory function[19]. Therefore, mutations in these domains may produce significant influences on JAK/STAT signaling pathway in the 8 patients.

MPN can be attributed to an acquired mutation such as JAK2V617.MPN may also have a familial clustering characterization, and familial MPN patients may present different clinical types of MPN in a family[32]. Moreover, findings from a Swedish research group confirmed that the first degree relatives of MPN patients exhibited a 5-to 7-fold elevated risk[33], which support the presence of a susceptibility gene predisposing to PV, ET, PMF and possibly to CML, and raise the possibility that the acquired mutation is secondary to the genetic predisposition[34]. Therefore, several groups aimed to discover the predisposition gene and related single nucleotide polymorphisms among sporadic MPN and familial MPN [35, 36], but so far no direct evidence can explain these phenomena. Importantly, Our SNP study and mutation findings may offer a direct evidence to support genetic predisposition involve in the pathogenesis of MPN.

## Supporting Information

S1 File The list of the participant-level data on SNPs and mutations. (DOCX)
