# Supplementary material for: The Polymorphisms in LNK Gene Correlated to the Clinical Type of Myeloproliferative Neoplasms
Source: PLoS One. 2016 Apr 25;11(4):e0154183. doi: 10.1371/journal.pone.0154183 (PMC4844169; doi:10.1371/journal.pone.0154183)
Supplement: S1 File — (DOCX) [file pone.0154183.s001.docx]

| Number | Gender | Age | Type | JAK2 or BCR-ABL | Sample | rs3184504 | rs78894077 | rs111340708 |
| --- | --- | --- | --- | --- | --- | --- | --- | --- |
| 1 | Male | 49 | control |  | PB | ( CC ) | ( TT ) | x4 |
| 2 | Male | 51 | control |  | BM | ( CC ) | ( TT ) | x4 |
| 3 | Male | 53 | control |  | BM | ( CC ) | ( TT ) | x4 |
| 4 | Male | 51 | control |  | BM | ( CC ) | ( TT ) | x4 |
| 5 | Male | 42 | control |  | PB | ( CC ) | ( TT ) | x4 |
| 6 | Male | 53 | control |  | PB | ( CC ) | ( TT ) | x4 |
| 7 | Male | 34 | control |  | BM | ( CC ) | ( TT ) | x4 |
| 8 | Female | 38 | control |  | PB | ( CC ) | ( TT ) | x4 |
| 9 | Female | 34 | control |  | PB | ( CC ) | ( TT ) | x4 |
| 10 | Male | 53 | control |  | BM | ( CC ) | ( TT ) | x4 |
| 11 | Male | 42 | control |  | BM | ( CC ) | ( TT ) | x4 |
| 12 | Male | 42 | control |  | BM | ( CC ) | ( TT ) | x4 |
| 13 | Female | 39 | control |  | BM | ( CC ) | ( TT ) | x4 |
| 14 | Female | 38 | control |  | PB | ( CC ) | ( TT ) | x4 |
| 15 | Female | 34 | control |  | PB | ( CC ) | ( TT ) | x4 |
| 16 | Male | 35 | control |  | BM | ( CC ) | ( TT ) | x4 |
| 17 | Male | 41 | control |  | PB | ( CC ) | ( TT ) | x4 |
| 18 | Male | 53 | control |  | PB | ( CC ) | ( TT ) | x5/x4 |
| 19 | Male | 44 | control |  | PB | ( CC ) | ( TT ) | x5/x4 |
| 20 | Female | 35 | control |  | PB | ( CC ) | ( TT ) | x5/x4 |
| 21 | Male | 41 | control |  | PB | ( CC ) | ( TT ) | x5/x4 |
| 22 | Male | 32 | control |  | PB | ( CC ) | ( TT ) | x5/x4 |
| 23 | Female | 42 | control |  | PB | ( CC ) | ( TT ) | x5 |
| 24 | Female | 29 | control |  | PB | ( CC ) | ( TT ) | x5 |
| 25 | Female | 33 | control |  | PB | ( CC ) | ( TT ) | x5 |
| 26 | Male | 39 | control |  | PB | ( CC ) | ( TT ) | x5 |
| 27 | Female | 49 | control |  | BM | ( CC ) | ( TT ) | x5 |
| 28 | Female | 28 | control |  | BM | ( CC ) | ( TT ) | x5 |
| 29 | Male | 52 | control |  | PB | ( CC ) | ( TT ) | x5 |
| 30 | Male | 27 | control |  | BM | ( CC ) | ( TT ) | x5 |
| 31 | Male | 39 | control |  | BM | ( CC ) | ( TT ) | x5 |
| 32 | Female | 45 | control |  | PB | ( CC ) | ( TT ) | x5 |
| 33 | Male | 24 | control |  | BM | ( CC ) | ( TC ) | x4 |
| 34 | Female | 33 | control |  | PB | ( CC ) | ( TC ) | x4 |
| 35 | Male | 33 | control |  | PB | ( CC ) | ( TC ) | x4 |
| 36 | Female | 34 | control |  | BM | ( CC ) | ( TC ) | x4 |
| 37 | Female | 41 | control |  | PB | ( CC ) | ( TC ) | x5 |
| 38 | Male | 37 | control |  | BM | ( CC ) | ( CC ) | x4 |
| 39 | Female | 22 | control |  | PB | ( TT ) | ( TT ) | x4 |
| 40 | Female | 36 | control |  | PB | ( TT ) | ( TT ) | x4 |
| 41 | Female | 49 | control |  | PB | ( TT ) | ( TT ) | x4 |
| 42 | Male | 32 | control |  | PB | ( TT ) | ( TT ) | x4 |
| 43 | Male | 49 | control |  | PB | ( TT ) | ( TT ) | x4 |
| 44 | Female | 43 | control |  | BM | ( TT ) | ( TT ) | x4 |
| 45 | Female | 29 | control |  | BM | ( TT ) | ( TT ) | x4 |
| 46 | Female | 43 | control |  | BM | ( TT ) | ( TT ) | x4 |
| 47 | Male | 42 | control |  | PB | ( TT ) | ( TT ) | x4 |
| 48 | Female | 29 | control |  | BM | ( TT ) | ( TT ) | x4 |
| 49 | Male | 28 | control |  | BM | ( TT ) | ( TT ) | x4 |
| 50 | Male | 53 | control |  | BM | ( TT ) | ( TT ) | x4 |
| 51 | Male | 42 | control |  | PB | ( TT ) | ( TT ) | x4 |
| 52 | Male | 32 | control |  | PB | ( TT ) | ( TT ) | x5 |
| 53 | Male | 20 | control |  | PB | ( TT ) | ( TT ) | x5 |
| 54 | Female | 29 | control |  | PB | ( TT ) | ( TT ) | x5 |
| 55 | Male | 32 | control |  | PB | ( TT ) | ( TT ) | x5 |
| 56 | Male | 52 | control |  | PB | ( TT ) | ( TT ) | x5 |
| 57 | Female | 49 | control |  | PB | ( TT ) | ( TT ) | x5 |
| 58 | Female | 31 | control |  | PB | ( TT ) | ( TT ) | x5 |
| 59 | Male | 43 | control |  | PB | ( TT ) | ( TT ) | x5 |
| 60 | Female | 51 | control |  | BM | ( TT ) | ( TT ) | x5 |
| 61 | Female | 32 | control |  | BM | ( TT ) | ( TT ) | x5 |
| 62 | Male | 33 | control |  | BM | ( TT ) | ( TT ) | x5 |
| 63 | Male | 24 | control |  | BM | ( TT ) | ( TT ) | x5 |
| 64 | Female | 40 | control |  | BM | ( TT ) | ( TT ) | x5 |
| 65 | Female | 31 | control |  | BM | ( TT ) | ( TT ) | x5 |
| 66 | Male | 45 | control |  | PB | ( TT ) | ( TT ) | x5 |
| 67 | Female | 31 | control |  | PB | ( TT ) | ( TT ) | x5 |
| 68 | Male | 32 | control |  | PB | ( TT ) | ( TT ) | x5 |
| 69 | Female | 42 | control |  | PB | ( TT ) | ( TT ) | x5 |
| 70 | Male | 40 | control |  | PB | ( TT ) | ( TT ) | x5/x4 |
| 71 | Female | 49 | control |  | PB | ( TT ) | ( TT ) | x5/x4 |
| 72 | Female | 45 | control |  | PB | ( TT ) | ( TT ) | x5/x4 |
| 73 | Male | 40 | control |  | PB | ( TT ) | ( TC ) | x4 |
| 74 | Female | 32 | control |  | PB | ( TT ) | ( TC ) | x4 |
| 75 | Male | 28 | control |  | BM | ( TT ) | ( TC ) | x4 |
| 76 | Male | 34 | control |  | BM | ( TT ) | ( TC ) | x5 |
| 77 | Male | 45 | control |  | PB | ( TC ) | ( TT ) | x4 |
| 78 | Male | 29 | control |  | PB | ( TC ) | ( TT ) | x4 |
| 79 | Female | 42 | control |  | PB | ( TC ) | ( TT ) | x4 |
| 80 | Male | 33 | control |  | PB | ( TC ) | ( TT ) | x5 |
| 81 | Female | 53 | control |  | PB | ( TC ) | ( TT ) | x5 |
| 82 | Female | 49 | control |  | PB | ( TC ) | ( TT ) | x5 |
| 83 | Female | 53 | control |  | PB | ( TC ) | ( TT ) | x5 |
| 84 | Female | 43 | control |  | PB | ( TC ) | ( TT ) | x5 |
| 85 | Male | 38 | control |  | PB | ( TC ) | ( TT ) | x5 |
| 86 | Female | 35 | control |  | BM | ( TC ) | ( TT ) | x5 |
| 87 | Male | 33 | control |  | PB | ( TC ) | ( TC ) | x4 |
| 88 | Male | 29 | control |  | PB | ( TC ) | ( TC ) | x4 |
| 89 | Male | 51 | control |  | BM | ( TC ) | ( TC ) | x4 |
| 90 | Male | 42 | control |  | BM | ( TC ) | ( TC ) | x4 |
| 91 | Male | 29 | control |  | BM | ( TC ) | ( TC ) | x5/x4 |
| 92 | Male | 31 | control |  | BM | ( TC ) | ( TC ) | x5/x4 |
| 93 | Male | 43 | control |  | BM | ( TC ) | ( TC ) | x5 |
| 94 | Male | 62 | PV | (-) | PB | ( CC ) | ( TT ) | x5 |
| 95 | Female | 28 | PV | (+) | BM | （ CC ） | ( TT ) | x4 |
| 96 | Female | 61 | PV | (+) | BM | ( CC ) | ( TT ) | x4 |
| 97 | Female | 49 | PV | (+) | BM | ( CC ) | ( TT ) | x4 |
| 98 | Female | 52 | PV | (+) | BM | ( CC ) | ( TT ) | x4 |
| 99 | Female | 41 | PV | (+) | BM | ( CC ) | ( TT ) | x4 |
| 100 | Female | 58 | PV | (+) | BM | ( CC ) | ( TT ) | x4 |
| 101 | Male | 58 | PV | (-) | BM | ( CC ) | ( TC ) | x4 |
| 102 | Male | 38 | PV | (-) | BM | ( CC ) | ( TC ) | x4 |
| 103 | Female | 73 | PV | (+) | BM | ( CC ) | ( TT ) | x4 |
| 104 | Male | 72 | PV | (-) | PB | ( TT ) | ( TT ) | x5 |
| 105 | Male | 61 | PV | (-) | BM | ( TT ) | ( TT ) | x5 |
| 106 | Male | 67 | PV | (-) | PB | ( TT ) | ( TT ) | x5 |
| 107 | Male | 52 | PV | (-) | BM | ( TT ) | ( TT ) | x5 |
| 108 | Female | 41 | PV | (+) | BM | ( TT ) | ( TT ) | x4 |
| 109 | Male | 72 | PV | (+) | PB | ( TT ) | ( TT ) | x4 |
| 110 | Male | 20 | PV | (+) | BM | ( TT ) | ( TT ) | x4 |
| 111 | Female | 54 | PV | (+) | BM | ( TT ) | ( TT ) | x4 |
| 112 | Male | 36 | PV | (+) | BM | ( TT ) | ( TT ) | x4 |
| 113 | Male | 60 | PV | (+) | BM | ( TT ) | ( TT ) | x4 |
| 114 | Male | 59 | PV | (+) | BM | ( TT ) | ( TT ) | x4 |
| 115 | Female | 50 | PV | (+) | PB | ( TT ) | ( TT ) | x5/x4 |
| 116 | Male | 55 | PV | (+) | BM | ( TT ) | ( TT ) | x5/x4 |
| 117 | Male | 57 | PV | (+) | BM | ( TT ) | ( TT ) | x5/x4 |
| 118 | Female | 72 | PV | (+) | BM | ( TT ) | ( TT ) | x5/x4 |
| 119 | Female | 50 | PV | (+) | BM | ( TT ) | ( TT ) | x5/x4 |
| 120 | Male | 32 | PV | (+) | BM | ( TT ) | ( TT ) | x5/x4 |
| 121 | Male | 69 | PV | (+) | BM | ( TT ) | ( TT ) | x5/x4 |
| 122 | Male | 46 | PV | (+) | PB | ( TT ) | ( TT ) | x5/x4 |
| 123 | Male | 40 | PV | (+) | BM | ( TT ) | ( TT ) | x5/x4 |
| 124 | Male | 58 | PV | (+) | PB | ( TT ) | ( TT ) | x5/x4 |
| 125 | Male | 74 | PV | (+) | BM | ( TT ) | ( TT ) | x5/x4 |
| 126 | Female | 46 | PV | (+) | BM | ( TT ) | ( TT ) | x5/x4 |
| 127 | Male | 53 | PV | (+) | BM | ( TT ) | ( TT ) | x5/x4 |
| 128 | Female | 72 | PV | (+) | PB | ( TT ) | ( TT ) | x5/x4 |
| 129 | Female | 41 | PV | (+) | BM | ( TT ) | ( TT ) | x5/x4 |
| 130 | Male | 30 | PV | (+) | BM | ( TT ) | ( TT ) | x5/x4 |
| 131 | Male | 45 | PV | (+) | BM | ( TT ) | ( TT ) | x5/x4 |
| 132 | Male | 29 | PV | (+) | BM | ( TT ) | ( TT ) | x5/x4 |
| 133 | Male | 49 | PV | (+) | BM | ( TT ) | ( TT ) | x5/x4 |
| 134 | Male | 28 | PV | (+) | BM | ( TT ) | ( TT ) | x5/x4 |
| 135 | Female | 78 | PV | (+) | PB | ( TT ) | ( TT ) | x5/x4 |
| 136 | Female | 34 | PV | (+) | BM | ( TT ) | ( TT ) | x5/x4 |
| 137 | Female | 63 | PV | (+) | BM | ( TT ) | ( TT ) | x5/x4 |
| 138 | Female | 58 | PV | (+) | BM | ( TT ) | ( TT ) | x5/x4 |
| 139 | Female | 44 | PV | (+) | BM | ( TT ) | ( TT ) | x5/x4 |
| 140 | Male | 48 | PV | (+) | BM | ( TT ) | ( TT ) | x5 |
| 141 | Female | 55 | PV | (+) | BM | ( TT ) | ( TT ) | x5 |
| 142 | Female | 68 | PV | (+) | BM | ( TT ) | ( TT ) | x5 |
| 143 | Male | 65 | PV | (+) | BM | ( TT ) | ( TT ) | x5 |
| 144 | Male | 69 | PV | (+) | PB | ( TT ) | ( TT ) | x5 |
| 145 | Male | 31 | PV | (+) | PB | ( TT ) | ( TT ) | x5 |
| 146 | Male | 42 | PV | (+) | BM | ( TT ) | ( TT ) | x5 |
| 147 | Female | 72 | PV | (+) | BM | ( TT ) | ( TT ) | x5 |
| 148 | Male | 62 | PV | (+) | PB | ( TT ) | ( TT ) | x5 |
| 149 | Female | 54 | PV | (+) | PB | ( TT ) | ( TT ) | x5 |
| 150 | Male | 69 | PV | (+) | BM | ( TT ) | ( TT ) | x5 |
| 151 | Male | 71 | PV | (+) | BM | ( TT ) | ( TT ) | x5 |
| 152 | Female | 66 | PV | (+) | BM | ( TT ) | ( TT ) | x5 |
| 153 | Female | 72 | PV | (+) | BM | ( TT ) | ( TT ) | x5 |
| 154 | Male | 73 | PV | (+) | BM | ( TT ) | ( TT ) | x5 |
| 155 | Female | 58 | PV | (+) | PB | ( TT ) | ( TT ) | x5 |
| 156 | Male | 72 | PV | (+) | BM | ( TT ) | ( TT ) | x5 |
| 157 | Male | 70 | PV | (+) | BM | ( TT ) | ( TT ) | x5 |
| 158 | Male | 40 | PV | (+) | PB | ( TT ) | ( TT ) | x5 |
| 159 | Male | 72 | PV | (+) | BM | ( TT ) | ( TC ) | x4 |
| 160 | Female | 68 | PV | (+) | BM | ( TT ) | ( TC ) | x4 |
| 161 | Male | 56 | PV | (+) | BM | ( TT ) | ( TC ) | x4 |
| 162 | Male | 61 | PV | (+) | BM | ( TT ) | ( TC ) | x4 |
| 163 | Male | 56 | PV | (+) | BM | ( TT ) | ( TC ) | x4 |
| 164 | Female | 47 | PV | (+) | BM | ( TT ) | ( TC ) | x4 |
| 165 | Male | 38 | PV | (+) | BM | ( TT ) | ( TC ) | x4 |
| 166 | Female | 66 | PV | (+) | PB | ( TT ) | ( TC ) | x5 |
| 167 | Female | 73 | PV | (+) | PB | ( TT ) | ( TC ) | x5 |
| 168 | Male | 50 | PV | (+) | BM | ( TT ) | ( TC ) | x5 |
| 169 | Male | 68 | PV | (+) | BM | ( TT ) | ( TC ) | x5 |
| 170 | Male | 23 | ET | (-) | BM | ( CC ) | ( TT ) | x4 |
| 171 | Female | 36 | ET | (-) | BM | ( CC ) | ( TT ) | x4 |
| 172 | Female | 61 | ET | (-) | BM | ( CC ) | ( TT ) | x4 |
| 173 | Female | 54 | ET | (-) | BM | ( CC ) | ( TT ) | x4 |
| 174 | Female | 71 | ET | (-) | BM | ( CC ) | ( TT ) | x4 |
| 175 | Female | 51 | ET | (-) | PB | ( CC ) | ( TT ) | x4 |
| 176 | Male | 63 | ET | (-) | BM | ( CC ) | ( TT ) | x4 |
| 177 | Female | 37 | ET | (-) | BM | ( CC ) | ( TT ) | x5/x4 |
| 178 | Female | 36 | ET | (+) | BM | ( CC ) | ( TT ) | x5/x4 |
| 179 | Female | 50 | ET | (+) | BM | ( CC ) | ( TT ) | x5/x4 |
| 180 | Female | 73 | ET | (+) | PB | ( CC ) | ( TT ) | x5/x4 |
| 181 | Female | 53 | ET | (+) | PB | ( CC ) | ( TT ) | x5/x4 |
| 182 | Female | 39 | ET | (+) | BM | ( CC ) | ( TT ) | x5 |
| 183 | Female | 35 | ET | (+) | BM | ( CC ) | ( TT ) | x5 |
| 184 | Female | 25 | ET | (+) | BM | ( CC ) | ( TT ) | x5 |
| 185 | Female | 81 | ET | (+) | BM | ( CC ) | ( TT ) | x5 |
| 186 | Female | 45 | ET | (+) | BM | ( CC ) | ( TT ) | x5 |
| 187 | Male | 22 | ET | (+) | BM | ( CC ) | ( TC ) | x5 |
| 188 | Female | 19 | ET | (-) | PB | ( TT ) | ( TT ) | x4 |
| 189 | Female | 70 | ET | (-) | PB | ( TT ) | ( TT ) | x4 |
| 190 | Female | 54 | ET | (-) | BM | ( TT ) | ( TT ) | x4 |
| 191 | Male | 59 | ET | (-) | BM | ( TT ) | ( TT ) | x4 |
| 192 | Male | 41 | ET | (-) | BM | ( TT ) | ( TT ) | x4 |
| 193 | Female | 46 | ET | (-) | BM | ( TT ) | ( TT ) | x4 |
| 194 | Male | 58 | ET | (-) | BM | ( TT ) | ( TT ) | x4 |
| 195 | Female | 73 | ET | (-) | BM | ( TT ) | ( TT ) | x4 |
| 196 | Female | 38 | ET | (-) | BM | ( TT ) | ( TT ) | x4 |
| 197 | Male | 39 | ET | (-) | PB | ( TT ) | ( TT ) | x4 |
| 198 | Female | 23 | ET | (-) | BM | ( TT ) | ( TT ) | x4 |
| 199 | Female | 58 | ET | (-) | PB | ( TT ) | ( TT ) | x4 |
| 200 | Male | 13 | ET | (-) | PB | ( TT ) | ( TT ) | x4 |
| 201 | Female | 72 | ET | (-) | BM | ( TT ) | ( TT ) | x4 |
| 202 | Female | 33 | ET | (-) | BM | ( TT ) | ( TT ) | x4 |
| 203 | Male | 62 | ET | (-) | PB | ( TT ) | ( TT ) | x4 |
| 204 | Male | 51 | ET | (-) | PB | ( TT ) | ( TT ) | x4 |
| 205 | Female | 67 | ET | (-) | BM | ( TT ) | ( TT ) | x4 |
| 206 | Female | 35 | ET | (-) | BM | ( TT ) | ( TT ) | x4 |
| 207 | Female | 42 | ET | (-) | BM | ( TT ) | ( TT ) | x4 |
| 208 | Male | 45 | ET | (-) | PB | ( TT ) | ( TT ) | x5/x4 |
| 209 | Male | 43 | ET | (-) | BM | ( TT ) | ( TT ) | x5/x4 |
| 210 | Male | 54 | ET | (-) | BM | ( TT ) | ( TT ) | x5/x4 |
| 211 | Female | 68 | ET | (-) | BM | ( TT ) | ( TT ) | x5/x4 |
| 212 | Male | 48 | ET | (-) | BM | ( TT ) | ( TT ) | x5/x4 |
| 213 | Female | 72 | ET | (-) | PB | ( TT ) | ( TT ) | x5/x4 |
| 214 | Male | 68 | ET | (-) | BM | ( TT ) | ( TT ) | x5/x4 |
| 215 | Female | 40 | ET | (-) | PB | ( TT ) | ( TT ) | x5/x4 |
| 216 | Female | 36 | ET | (-) | PB | ( TT ) | ( TT ) | x5/x4 |
| 217 | Male | 70 | ET | (-) | BM | ( TT ) | ( TT ) | x5/x4 |
| 218 | Female | 72 | ET | (-) | BM | ( TT ) | ( TT ) | x5/x4 |
| 219 | Female | 34 | ET | (-) | BM | ( TT ) | ( TT ) | x5/x4 |
| 220 | Female | 55 | ET | (-) | BM | ( TT ) | ( TT ) | x5/x4 |
| 221 | Male | 59 | ET | (-) | BM | ( TT ) | ( TT ) | x5/x4 |
| 222 | Male | 47 | ET | (-) | BM | ( TT ) | ( TT ) | x5 |
| 223 | Male | 46 | ET | (-) | PB | ( TT ) | ( TT ) | x5 |
| 224 | Female | 70 | ET | (-) | BM | ( TT ) | ( TT ) | x5 |
| 225 | Female | 40 | ET | (-) | PB | ( TT ) | ( TT ) | x5 |
| 226 | Female | 70 | ET | (-) | BM | ( TT ) | ( TT ) | x5 |
| 227 | Male | 58 | ET | (-) | BM | ( TT ) | ( TT ) | x5 |
| 228 | Female | 31 | ET | (-) | BM | ( TT ) | ( TT ) | x5 |
| 229 | Female | 34 | ET | (-) | BM | ( TT ) | ( TT ) | x5 |
| 230 | Male | 28 | ET | (-) | PB | ( TT ) | ( TT ) | x5 |
| 231 | Male | 42 | ET | (-) | BM | ( TT ) | ( TT ) | x5 |
| 232 | Male | 46 | ET | (-) | PB | ( TT ) | ( TT ) | x5 |
| 233 | Male | 70 | ET | (-) | PB | ( TT ) | ( TT ) | x5 |
| 234 | Male | 72 | ET | (-) | BM | ( TT ) | ( TT ) | x5 |
| 235 | Male | 56 | ET | (-) | BM | ( TT ) | ( TT ) | x5 |
| 236 | Male | 66 | ET | (-) | PB | ( TT ) | ( TT ) | x5 |
| 237 | Male | 67 | ET | (-) | BM | ( TT ) | ( TT ) | x5 |
| 238 | Female | 71 | ET | (-) | BM | ( TT ) | ( TT ) | x5 |
| 239 | Male | 73 | ET | (-) | PB | ( TT ) | ( TT ) | x5 |
| 240 | Male | 72 | ET | (-) | BM | ( TT ) | ( TT ) | x5 |
| 241 | Female | 17 | ET | (-) | PB | ( TT ) | ( TT ) | x5 |
| 242 | Male | 24 | ET | (-) | PB | ( TT ) | ( TT ) | x5 |
| 243 | Female | 43 | ET | (-) | PB | ( TT ) | ( TT ) | x5 |
| 244 | Female | 28 | ET | (-) | BM | ( TT ) | ( TT ) | x5 |
| 245 | Female | 35 | ET | (-) | BM | ( TT ) | ( TT ) | x5 |
| 246 | Female | 25 | ET | (-) | PB | ( TT ) | ( TT ) | x5 |
| 247 | Female | 72 | ET | (+) | BM | ( TT ) | ( TT ) | x4 |
| 248 | Female | 51 | ET | (+) | BM | ( TT ) | ( TT ) | x4 |
| 249 | Male | 72 | ET | (+) | BM | ( TT ) | ( TT ) | x5 |
| 250 | Female | 32 | ET | (+) | BM | ( TT ) | ( TT ) | x5 |
| 251 | Female | 8 | ET | (+) | PB | ( TT ) | ( TT ) | x5 |
| 252 | Male | 69 | ET | (+) | BM | ( TT ) | ( TT ) | x5 |
| 253 | Female | 51 | ET | (+) | PB | ( TT ) | ( TT ) | x5 |
| 254 | Female | 60 | ET | (+) | BM | ( TT ) | ( TT ) | x5 |
| 255 | Female | 59 | ET | (+) | BM | ( TT ) | ( TT ) | x5 |
| 256 | Female | 68 | ET | (+) | BM | ( TT ) | ( TT ) | x5 |
| 257 | Male | 66 | ET | (+) | BM | ( TT ) | ( TT ) | x5 |
| 258 | Female | 49 | ET | (+) | BM | ( TT ) | ( TT ) | x5 |
| 259 | Male | 72 | ET | (+) | BM | ( TT ) | ( TT ) | x5 |
| 260 | Female | 56 | ET | (+) | BM | ( TT ) | ( TT ) | x5 |
| 261 | Female | 72 | ET | (+) | BM | ( TT ) | ( TT ) | x5 |
| 262 | Male | 61 | ET | (+) | BM | ( TT ) | ( TT ) | x5 |
| 263 | Male | 58 | ET | (+) | PB | ( TT ) | ( TT ) | x5 |
| 264 | Male | 72 | ET | (+) | BM | ( TT ) | ( TT ) | x5 |
| 265 | Female | 71 | ET | (+) | BM | ( TT ) | ( TT ) | x5 |
| 266 | Male | 69 | ET | (+) | PB | ( TT ) | ( TT ) | x5 |
| 267 | Female | 72 | ET | (+) | BM | ( TT ) | ( TT ) | x5 |
| 268 | Female | 48 | ET | (+) | BM | ( TT ) | ( TT ) | x5 |
| 269 | Female | 49 | ET | (+) | BM | ( TT ) | ( TT ) | x5 |
| 270 | Female | 45 | ET | (+) | BM | ( TT ) | ( TT ) | x5 |
| 271 | Female | 71 | ET | (+) | BM | ( TT ) | ( TT ) | x5 |
| 272 | Male | 72 | ET | (+) | BM | ( TT ) | ( TT ) | x5 |
| 273 | Female | 62 | ET | (+) | BM | ( TT ) | ( TT ) | x5 |
| 274 | Male | 29 | ET | (+) | PB | ( TT ) | ( TT ) | x5 |
| 275 | Female | 73 | ET | (+) | BM | ( TT ) | ( TT ) | x5 |
| 276 | Female | 59 | ET | (+) | BM | ( TT ) | ( TT ) | x5 |
| 277 | Female | 53 | ET | (+) | BM | ( TT ) | ( TT ) | x5 |
| 278 | Male | 42 | ET | (+) | BM | ( TT ) | ( TT ) | x5 |
| 279 | Female | 72 | ET | (+) | BM | ( TT ) | ( TT ) | x5 |
| 280 | Female | 59 | ET | (+) | BM | ( TT ) | ( TT ) | x5 |
| 281 | Male | 58 | ET | (+) | PB | ( TT ) | ( TT ) | x5 |
| 282 | Male | 34 | ET | (+) | BM | ( TT ) | ( TT ) | x5 |
| 283 | Male | 28 | ET | (+) | PB | ( TT ) | ( TT ) | x5 |
| 284 | Female | 53 | ET | (+) | BM | ( TT ) | ( TT ) | x5 |
| 285 | Female | 21 | ET | (+) | BM | ( TT ) | ( TT ) | x5 |
| 286 | Female | 72 | ET | (+) | BM | ( TT ) | ( TT ) | x5 |
| 287 | Female | 63 | ET | (+) | PB | ( TT ) | ( TT ) | x5 |
| 288 | Female | 71 | ET | (+) | BM | ( TT ) | ( TT ) | x5 |
| 289 | Male | 69 | ET | (+) | PB | ( TT ) | ( TT ) | x5 |
| 290 | Female | 61 | ET | (+) | BM | ( TT ) | ( TT ) | x5 |
| 291 | Female | 53 | ET | (+) | BM | ( TT ) | ( TT ) | x5 |
| 292 | Female | 51 | ET | (+) | BM | ( TT ) | ( TT ) | x5 |
| 293 | Female | 51 | ET | (+) | PB | ( TT ) | ( TT ) | x5 |
| 294 | Female | 40 | ET | (+) | BM | ( TT ) | ( TT ) | x5 |
| 295 | Female | 70 | ET | (+) | BM | ( TT ) | ( TT ) | x5 |
| 296 | Female | 74 | ET | (+) | BM | ( TT ) | ( TT ) | x5 |
| 297 | Male | 59 | ET | (+) | BM | ( TT ) | ( TT ) | x5 |
| 298 | Female | 55 | ET | (+) | PB | ( TT ) | ( TT ) | x5 |
| 299 | Male | 42 | ET | (+) | BM | ( TT ) | ( TT ) | x5 |
| 300 | Female | 64 | ET | (+) | BM | ( TT ) | ( TT ) | x5 |
| 301 | Female | 57 | ET | (+) | BM | ( TT ) | ( TT ) | x5 |
| 302 | Male | 68 | ET | (+) | BM | ( TT ) | ( TT ) | x5 |
| 303 | Male | 74 | ET | (+) | BM | ( TT ) | ( TT ) | x5 |
| 304 | Female | 58 | ET | (+) | BM | ( TT ) | ( TT ) | x5 |
| 305 | Female | 62 | ET | (+) | BM | ( TT ) | ( TT ) | x5 |
| 306 | Male | 57 | ET | (+) | BM | ( TT ) | ( TT ) | x5 |
| 307 | Male | 67 | ET | (+) | BM | ( TT ) | ( TT ) | x5 |
| 308 | Female | 72 | ET | (+) | BM | ( TT ) | ( TT ) | x5 |
| 309 | Male | 72 | ET | (+) | BM | ( TT ) | ( TT ) | x5 |
| 310 | Female | 68 | ET | (+) | BM | ( TT ) | ( TT ) | x5 |
| 311 | Female | 43 | ET | (-) | BM | ( TT ) | ( TC ) | x4 |
| 312 | Female | 66 | ET | (-) | BM | ( TT ) | ( TC ) | x5/x4 |
| 313 | Male | 72 | ET | (-) | BM | ( TT ) | ( TC ) | x5/x4 |
| 314 | Male | 73 | ET | (-) | BM | ( TT ) | ( TC ) | x5/x4 |
| 315 | Female | 68 | ET | (+) | BM | ( TT ) | ( TC ) | x4 |
| 316 | Male | 57 | ET | (+) | PB | ( TT ) | ( TC ) | x4 |
| 317 | Female | 50 | ET | (+) | BM | ( TT ) | ( TC ) | x5 |
| 318 | Female | 62 | ET | (+) | BM | ( TT ) | ( TC ) | x5 |
| 319 | Male | 63 | ET | (+) | BM | ( TT ) | ( TC ) | x5 |
| 320 | Female | 48 | ET | (+) | BM | ( TT ) | ( TC ) | x5 |
| 321 | Male | 33 | ET | (+) | BM | ( TT ) | ( TC ) | x5 |
| 322 | Female | 65 | ET | (+) | BM | ( TT ) | ( TC ) | x5 |
| 323 | Male | 73 | ET | (+) | BM | ( TT ) | ( TC ) | x5 |
| 324 | Female | 50 | PMF | (-) | PB | ( CC ) | ( TT ) | x5 |
| 325 | Female | 50 | PMF | (-) | BM | ( CC ) | ( TT ) | x5 |
| 326 | Female | 72 | PMF | (-) | BM | ( CC ) | ( TT ) | x5 |
| 327 | Male | 60 | PMF | (+) | PB | ( CC ) | ( TC ) | x5 |
| 328 | Male | 63 | PMF | (-) | BM | ( TT ) | ( TT ) | x4 |
| 329 | Female | 60 | PMF | (-) | BM | ( TT ) | ( TT ) | x5/x4 |
| 330 | Male | 29 | PMF | (-) | BM | ( TT ) | ( TT ) | x5 |
| 331 | Male | 58 | PMF | (-) | BM | ( TT ) | ( TT ) | x5 |
| 332 | Female | 53 | PMF | (-) | PB | ( TT ) | ( TT ) | x5 |
| 333 | Female | 73 | PMF | (-) | BM | ( TT ) | ( TT ) | x5 |
| 334 | Female | 31 | PMF | (-) | PB | ( TT ) | ( TT ) | x5 |
| 335 | Male | 44 | PMF | (-) | BM | ( TT ) | ( TT ) | x5 |
| 336 | Female | 46 | PMF | (+) | BM | ( TT ) | ( TT ) | x4 |
| 337 | Male | 21 | PMF | (+) | BM | ( TT ) | ( TT ) | x5/x4 |
| 338 | Female | 46 | PMF | (+) | BM | ( TT ) | ( TT ) | x5/x4 |
| 339 | Male | 72 | PMF | (+) | BM | ( TT ) | ( TT ) | x5/x4 |
| 340 | Male | 72 | PMF | (+) | BM | ( TT ) | ( TT ) | x5 |
| 341 | Male | 47 | PMF | (+) | BM | ( TT ) | ( TT ) | x5 |
| 342 | Female | 64 | PMF | (+) | BM | ( TT ) | ( TC ) | x5 |
| 343 | Male | 58 | CML | BCL/ABL | BM | ( CC ) | ( TT ) | x4 |
| 344 | Male | 31 | CML | BCL/ABL | BM | ( CC ) | ( TT ) | x4 |
| 345 | Male | 42 | CML | BCL/ABL | BM | ( CC ) | ( TT ) | x4 |
| 346 | Male | 69 | CML | BCL/ABL | BM | ( CC ) | ( TT ) | x4 |
| 347 | Female | 21 | CML | BCL/ABL | BM | ( CC ) | ( TT ) | x4 |
| 348 | Male | 20 | CML | BCL/ABL | BM | ( CC ) | ( TT ) | x5/x4 |
| 349 | Male | 58 | CML | BCL/ABL | BM | ( CC ) | ( TT ) | x5/x4 |
| 350 | Male | 38 | CML | BCL/ABL | BM | ( CC ) | ( TT ) | x5/x4 |
| 351 | Female | 74 | CML | BCL/ABL | BM | ( CC ) | ( TT ) | x5/x4 |
| 352 | Female | 53 | CML | BCL/ABL | PB | ( CC ) | ( TT ) | x5/x4 |
| 353 | Male | 42 | CML | BCL/ABL | BM | ( CC ) | ( TT ) | x5/x4 |
| 354 | Male | 70 | CML | BCL/ABL | BM | ( CC ) | ( TT ) | x5/x4 |
| 355 | Female | 34 | CML | BCL/ABL | BM | ( CC ) | ( TT ) | x5 |
| 356 | Male | 46 | CML | BCL/ABL | BM | ( CC ) | ( TT ) | x5 |
| 357 | Male | 70 | CML | BCL/ABL | BM | ( CC ) | ( TT ) | x5 |
| 358 | Male | 33 | CML | BCL/ABL | BM | ( CC ) | ( TT ) | x5 |
| 359 | Female | 38 | CML | BCL/ABL | BM | ( CC ) | ( TT ) | x5 |
| 360 | Male | 40 | CML | BCL/ABL | BM | ( CC ) | ( TT ) | x5 |
| 361 | Female | 28 | CML | BCL/ABL | BM | ( CC ) | ( TT ) | x5 |
| 362 | Female | 53 | CML | BCL/ABL | BM | ( CC ) | ( TT ) | x5 |
| 363 | Female | 42 | CML | BCL/ABL | BM | ( CC ) | ( TT ) | x5 |
| 364 | Male | 73 | CML | BCL/ABL | BM | ( CC ) | ( TT ) | x5 |
| 365 | Female | 21 | CML | BCL/ABL | BM | ( CC ) | ( TT ) | x5 |
| 366 | Female | 66 | CML | BCL/ABL | BM | ( CC ) | ( TT ) | x5 |
| 367 | Male | 40 | CML | BCL/ABL | BM | ( CC ) | ( TT ) | x5 |
| 368 | Male | 55 | CML | BCL/ABL | BM | ( CC ) | ( TT ) | x5 |
| 369 | Female | 34 | CML | BCL/ABL | BM | ( CC ) | ( TT ) | x5 |
| 370 | Male | 59 | CML | BCL/ABL | BM | ( CC ) | ( TT ) | x5 |
| 371 | Male | 21 | CML | BCL/ABL | BM | ( CC ) | ( CC ) | x4 |
| 372 | Male | 53 | CML | BCL/ABL | PB | ( CC ) | ( CC ) | x4 |
| 373 | Female | 28 | CML | BCL/ABL | BM | ( CC ) | ( CC ) | x4 |
| 374 | Female | 25 | CML | BCL/ABL | BM | ( CC ) | ( CC ) | x5/x4 |
| 375 | Male | 40 | CML | BCL/ABL | BM | ( CC ) | ( CC ) | x5/x4 |
| 376 | Female | 40 | CML | BCL/ABL | BM | ( CC ) | ( CC ) | x5/x4 |
| 377 | Female | 65 | CML | BCL/ABL | BM | ( TT ) | ( TT ) | x5 |
| 378 | Female | 58 | CML | BCL/ABL | BM | ( TT ) | ( TT ) | x5 |

| Number | Gender | Age | Type | JAK2 or BCR-ABL | Sample | rs3184504 | rs78894077 | rs111340708 |  |
| --- | --- | --- | --- | --- | --- | --- | --- | --- | --- |
| 247 | Female | 72 | ET | (+) | BM | ( TT ) | ( TT ) | x4 | A300V |
| 248 | Female | 51 | ET | (+) | BM | ( TT ) | ( TT ) | x4 | A300V |
| 316 | Male | 57 | ET | (+) | PB | ( TT ) | ( TC ) | x4 | A300V |
| 101 | Male | 58 | PV | (-) | BM | ( CC ) | ( TC ) | x4 | A300V |
| 359 | Female | 31 | CML | BCL/ABL | BM | ( CC ) | ( TT ) | x5 | A300V |
| 315 | Female | 68 | ET | (+) | BM | ( TT ) | ( TC ) | x4 | V402M |
| 336 | Female | 46 | PMF | (+) | BM | ( TT ) | ( TT ) | x4 | V402M |
| 140 | Male | 48 | PV | (+) | BM | ( TT ) | ( TT ) | x5 | R415H |

**Diagnostic criteria for Polycythemia Vera (PV).** Diagnosis requires the presence of both major criteria and one minor criterion or the presence of the first major criterion together with two minor criteria.

**Major criteria**

1.Haemoglobin>18.5g/dL in men, 16.5g/dL in women or other evidence of increased red cell volume.

2.Presence of JAK2V617F or other functionally similar mutation such as JAK2 exon 12 mutation.

**Minor criteria**

1.Bone marrow biopsy showing hyper-cellularity for age with trilineage growth (panmyelosis) with prominent erythrocyte, granulocytic and megakaryocytic proliferation.

2.Serum erythropoietin level below the reference range for normal.

3.Endogenous erythrocyte colony formation in vitro.

**Diagnostic criteria for essential thrombocythaemia (ET):** Diagnosis requires meeting all four criteria.

1.Sustained^a^ platelet count≥450x10^9^/L

2.Bone marrow biopsy specimen showing proliferation mainly of the megakaryocytic lineage with increased numbers of enlarged, mature megakaryocytes. No significant increase or left-shift of neutrophil granulopoiesis or erythropoiesis.

3.Not meeting WHO criteria for polycythemia vera ^b^, primary myelofibrosis ^c^, BCR-ABL1 positive chronic myelogenous leukemia ^d^ or myelodysplastic syndrome ^e^ or other myeloid neoplasm.

4. Demonstration of JAK2V617F or other clonal marker, or in the absence of JAK2V617F, no evidence for reactive thrombocytosis ^f^.

a. Sustained during the work-up process.

b. Requires the failure of iron replacement therapy to increase hemoglobin level to the polycythemia vera range in the presence of decreased serum ferritin. Exclusion of polycythemia vera is based on hemoglobin and hematocrit levels and red cell mass measurement is not required.

c. Requires the absence of relevant reticulin fibrosis, collagen fibrosis, peripheral blood leukoerythroblastosis, or markedly hypercellular marrow accompanied by megakaryocyte morphology that is typical for primary myelofibrosis including small to large megakaryocytes with an aberrant nuclear/cytoplasmic ratio and hyperchromatic, bulbous or irregularly folded nuclei and dense clustering.

d. Requires the absence of BCR-ABL1

e. Requires absence of dyserythropoiesis and dysgranulopoiesis.

f. Causes of reactive thrombocytosis include iron deficiency, splenectomy surgery, infection, inflammation, connective tissue disease, metastatic cancer, and lymphoproliferative disorders. However, the presence of a condition associated with reactive thrombocytosis may not exclude the possibility of ET if the first three criteria are met.

**Diagnostic criteria for primary myelofibrosis**: diagnosis requires meeting all 3 major and 2 minor criteria.

**Major criteria**

1.Presence of megakaryocyte proliferation and atypia ^a^, usually accompanied by either reticulin and/or collagen fibrosis, or in the absence of significant reticulin fibrosis, the megakaryocyte changes must be accompanied by an increased bone marrow cellularity characterized by granulocytic proliferation and often decreased erythropoiesis.

2. Not meeting WHO criteria for polycythemia vera ^b^, BCR-ABL1+chronic myelogenous leukemia ^c^, myelodysplastic syndrome ^d^, or other myeloid neoplasms.

3.Demonstration of JAK2V617F or other clonal marker (e.g. MPL W515K/L),

or

in the absence of a clonal marker, no evidence that the bone marrow fibrosis or other changes are secondary to infection, autoimmune disorder or other chronic inflammatory condition, hairy cell leukemia or other lymphoid neoplasm, metastatic malignancy, or toxic (chronic) myelopathies ^e^

**Minor criteria**

1.Leukoerythroblastosis^f^

2.Increase in serum lactate dehydrogenase level ^f^

3.Anaemia^f^

4.Splenomegaly^f^

a. Small to large megakaryocytes with an aberrant nuclear/cytoplasmic ratio and hyperchromatic, bulbous, or irregularly folded nuclei and dense clustering.

b. Requires the failure of iron replacement therapy to increase hemoglobin level to the polycythemia vera range in the presence of decreased serum ferritin. Exclusion of polycythemia vera is based on hemoglobin and hematocrit levels, and red cell mass measurement is not required.

c. Requires the absence of BCR-ABL1

d. Requires absence of dyserythropoiesis and dysgranulopoiesis

e. Patients with conditions associated with reactive myelofibrosis are not immune to PMF, and the diagnosis should be considered in such cases if other criteria are met.

f. Degree of abnormality could be borderline or marked.
